# Maternal immune response and air pollution exposure during pregnancy: insights from the Early Markers for Autism (EMA) study

**DOI:** 10.1186/s11689-020-09343-0

**Published:** 2020-12-16

**Authors:** Heather E. Volk, Bo Park, Calliope Hollingue, Karen L. Jones, Paul Ashwood, Gayle C. Windham, Fred Lurman, Stacey E. Alexeeff, Martin Kharrazi, Michelle Pearl, Judy Van de Water, Lisa A. Croen

**Affiliations:** 1 Department of Mental Health, Wendy Klag Center for Autism and Developmental Disabilities, Bloomberg School of Public Health, Johns Hopkins University, Kennedy Krieger Institute Intellectual and Developmental Disabilities Research Center, 624 N. Broadway, HH833, Baltimore, MD 21205 USA; 2 Department of Public Health, California State University, Fullerton, CA USA; 3 UC Davis MIND Institute, University of California Davis, Davis, CA USA; 4 Environmental Health Investigations Branch, California Department of Public Health, Richmond, CA USA; 5 Sonoma Technology, Inc., Petaluma, CA USA; 6 Division of Research, Kaiser Permanente of Northern California, Oakland, CA USA

**Keywords:** Air pollution, Immune response, Prenatal exposure, Intellectual disability, Autism spectrum disorder

## Abstract

**Background:**

Perinatal exposure to air pollution and immune system dysregulation are two factors consistently associated with autism spectrum disorders (ASD) and other neurodevelopmental outcomes. However, little is known about how air pollution may influence maternal immune function during pregnancy.

**Objectives:**

To assess the relationship between mid-gestational circulating levels of maternal cytokines/chemokines and previous month air pollution exposure across neurodevelopmental groups, and to assess whether cytokines/chemokines mediate the relationship between air pollution exposures and risk of ASD and/or intellectual disability (ID) in the Early Markers for Autism (EMA) study.

**Methods:**

EMA is a population-based, nested case–control study which linked archived maternal serum samples collected during weeks 15–19 of gestation for routine prenatal screening, birth records, and Department of Developmental Services (DDS) records. Children receiving DDS services for ASD without intellectual disability (ASD without ID; *n* = 199), ASD with ID (ASD with ID; *n* = 180), ID without ASD (ID; *n* = 164), and children from the general population (GP; *n* = 414) with no DDS services were included in this analysis. Serum samples were quantified for 22 cytokines/chemokines using Luminex multiplex analysis technology. Air pollution exposure for the month prior to maternal serum collection was assigned based on the Environmental Protection Agency’s Air Quality System data using the maternal residential address reported during the prenatal screening visit.

**Results:**

Previous month air pollution exposure and mid-gestational maternal cytokine and chemokine levels were significantly correlated, though weak in magnitude (ranging from − 0.16 to 0.13). Ten pairs of mid-pregnancy immune markers and previous month air pollutants were significantly associated within one of the child neurodevelopmental groups, adjusted for covariates (*p* < 0.001). Mid-pregnancy air pollution was not associated with any neurodevelopmental outcome. IL-6 remained associated with ASD with ID even after adjusting for air pollution exposure.

**Conclusion:**

This study suggests that maternal immune activation is associated with risk for neurodevelopmental disorders. Furthermore, that prenatal air pollution exposure is associated with small, but perhaps biologically relevant, effects on maternal immune system function during pregnancy. Additional studies are needed to better evaluate how prenatal exposure to air pollution affects the trajectory of maternal immune activation during pregnancy, if windows of heightened susceptibility can be identified, and how these factors influence neurodevelopment of the offspring.

**Supplementary Information:**

The online version contains supplementary material available at 10.1186/s11689-020-09343-0.

## Introduction

Air pollution is a common environmental exposure with known effects for a broad range of health outcomes [48]. In addition to reported associations with preterm birth, decreased birth weight, and smaller head circumference at birth (X [32, 37].), research on air pollution exposure during pregnancy suggests associations with altered neurodevelopmental outcomes [9, 13, 22] and specifically autism spectrum disorder (ASD) [8, 30]. These findings have generated substantial interest in delineating the potential mechanisms by which air pollution exposure can affect neurodevelopment during gestation and early life.

A separate literature shows that air pollution exposure induces an inflammatory response [27, 33, 40, 41]. Ambient air pollution, and particulate matter (PM) in particular, induces an innate immune response leading to activation of transcription factors and increased downstream proinflammatory cytokine production [36]. Results from epidemiologic studies support this, as prenatal air pollution has been shown to alter the immune response in the cord blood [20] [3].

Maternal immune activation during the prenatal period has been associated with atypical brain development [28]. Prenatal maternal immune activation through bacterial or viral infection has been associated with increased risk of ASD [4, 7, 31, 47]. Several studies have examined the relationship between maternal mid-gestational cytokines and chemokines and ASD. A previous study from our group found increased maternal mid-gestation serum levels of interferon gamma (IFNγ), IL-4, and IL-5 in 84 mothers of children diagnosed with ASD compared to mothers of children from the general population with no known neurodevelopmental disorders (P. E [16].). Previously, in the Early Markers for Autism (EMA) study, we observed increased levels of mid-gestational cytokines and chemokines among mothers of children with both ASD and intellectual disability (ID), strengthening the evidence for the role of gestational immune dysregulation and risk of neurodevelopmental disorders [23]. The different proinflammatory profiles between mothers of children with ASD with ID, ASD without ID, and ID alone also suggest different etiologic pathways may be involved across these phenotypes. A study by Abdallah et al. using the Danish Historic Birth Cohort bolsters the hypothesis that an inflammatory state during fetal development is linked with neurodevelopmental disorders. The authors found elevated levels of IL-4, IL-10, tumor necrosis factor (TNF)-α, and TNF-β in the amniotic fluid were associated with ASD, whereas elevated levels of IL-5 and IL-6 associated with other childhood psychiatric disorders [1].

To our knowledge, no study has directly examined how the connection between maternal immune system cytokines and air pollution exposure during pregnancy and if this response was associated with later neurodevelopmental outcomes. The present study had two goals: (1) to characterize the relationship between maternal cytokine and chemokine levels measured during mid-pregnancy and previous month (short term) air pollution exposure and (2) to evaluate if immune markers measured mid-pregnancy mediate an association between mid-pregnancy air pollutant exposure and child neurodevelopmental disorders.

## Methods

### Study description

The Early Markers for Autism (EMA) study is a case-control study designed to investigate prenatal environmental exposures, maternal and child genetic susceptibility, immunologic factors, and their interplay in risk for ASD. Study participants were drawn from the cohort of children born in Orange, San Diego, or Imperial Counties in Southern California from January 2000 to June 2003, who survived to age 1, and whose mothers participated in prenatal screening. Children with ASD as well as children with non-ASD ID of unknown etiology (e.g., IQ < 70, with no known genetic or other etiology) were identified by linkage to records of the California Department of Developmental Services (DDS). DDS operates a system of 21 Regional Centers (RC) that coordinate services for persons with developmental disabilities regardless of ability to pay or socioeconomic status. After excluding all past or current DDS/RC clients, general population (GP) controls were sampled from the birth certificate files, matched to ASD cases by sex, birth month, and birth year. The same control group was used for children with ID, who did not have matched GP controls selected.

### Diagnostic validation

Following a protocol initially developed by the CDC [45], trained medical record abstractors reviewed and abstracted detailed diagnostic and clinical data from Regional Center records for all children with ASD and with ID of unknown etiology. Final case status was determined by expert clinical review of the abstracted information, with an ASD case definition based on meeting DSM-IV criteria. Final classification of ID without ASD was based on standardized test score results found in records (with composite scores on cognitive and functional testing of < 70 defined as ID) in the absence of ASD symptoms. ASD cases were further classified according to the presence/absence of ID (defined as developmental/cognitive score and adaptive composite score < 70 as for ID, but in the presence of ASD symptoms). After expert review, the EMA sample consisted of 427 children with ASD (of which 184 also had ID), 192 with ID but not ASD, and 439 GP controls (total *N* = 1058).

### Maternal serum collection

Maternal mid-pregnancy specimens were retrieved from the California Department of Public Health’s Project Baby’s Breath prenatal screening specimen archives. The archive includes maternal serum and blood cell pellet specimens collected for routine expanded prenatal α-fetoprotein screening program (XAFP) screening (typically during 15–19 weeks of gestation). Maternal specimens were collected in serum separator tubes by obstetrical care service providers or laboratories and underwent XAFP testing within 7 days of collection at a central laboratory (median time = 3 days). After 1–2 days of refrigeration, leftover specimens were stored at − 20 °C. Consent for the XAFP screening program was obtained at the time of the test requisition, and privacy notifications stipulated that specimens and data from prenatal testing could be used for legitimate research purposes given appropriate institutional review board approval. Maternal specimens were packed on dry ice and shipped directly to the immunology laboratory (JVW) using overnight delivery, where they were stored at − 80 °C until their use in cytokine and chemokine measurements described below.

### Cytokine and chemokine measurement

Maternal mid-gestational serum concentrations of 22 cytokines and chemokines were determined using a commercially available Multiplex Bead-Based Kit (Milliplex MAP Human Cytokine/Chemokine Kit; Millipore, Billerica, MA, USA) in accordance with the kit-specific protocols provided by Millipore. The following cytokines and chemokines were measured: granulocyte macrophage colony-stimulating factor (GM-CSF), IFNγ, IL-1α, IL-1β, IL-2, IL-4, IL-6, IL-7, IL-8, IL-10, IL-12p40, IL-12p70, IL-13, IL-17, IFNγ-induced protein 10 (IP-10), monocyte chemotactic protein 1 (MCP-1), macrophage inflammatory protein (MIP)-1α, MIP-1β, TNF-α, eotaxin, soluble IL-2 receptor-α (sIL-2Rα), and IL-1 receptor antagonist (IL-1Rα). Briefly, 25 μl of serum was diluted 1:2 in sample buffer and incubated with fluorescently labeled capture antibody-coated beads in a 96-well filter bottomed plate on a plate shaker overnight at 4 °C. After incubation, the sample-bead mix was removed and the plate washed two times using a vacuum manifold [23]. Biotinylated detection antibodies were then added and incubated for 1 h at room temperature with shaking. The reaction mixture was detected by the addition of streptavidin–phycoerythrin and incubated on a plate shaker at room temperature for 30 min. Following a repeat of the washing step, beads were resuspended in sheath fluid for 5 min on the plate shaker. Plates were read on a Bio-Plex 200 system (Bio-Rad Laboratories, Hercules, CA, USA) and analyzed using Bio-Plex Manager software (Bio-Rad Laboratories) with a five-parameter model used to calculate final concentrations and values (expressed in pg ml^−1^). Reference samples were run on each plate to determine assay consistency, and all samples were run blinded to child developmental outcome.

### Air pollution exposure assignment

In this analysis, we used the maternal residence recorded on the state of California Test Request Form collected at the time of routine prenatal screening for patient communication and billing purposes. This address was used to assign average air pollution exposure for the month prior to the prenatal screening blood collection. When this address was not available, the address recorded on the birth certificate was used. Each address was standardized and geo-coded using the TeleAtlas US_Geo_2 database and software (Tele Atlas, Inc., Boston, CA, www.geocoded.com).

Air quality assignments for PM_2.5_, PM_10_, ozone (O_3_), and NO_2_ were derived from the US EPA’s Air Quality System (AQS) data (www.epa.gov/ttn/airs/airsaqs). Air quality data for the 30 days preceding the blood sample collection date (“previous month”) was downloaded from monitoring stations located within 50 km of each address; previous month average was calculated based on these daily levels. To assign exposure to each location, we used inverse distance-squared weighting (IDW2) of data from up to four closest stations located within 50 km of each participant residence. However, if one or more stations were located within 5 km of a residence, only data from those stations were used for the exposure assessment. Because studies have shown large offshore to onshore pollutant gradients along the southern California coast, the interpolations were carried out with pseudo-stations, or theoretical locations used for estimating pollution gradients from extant data when geography did not permit observed data, located ~ 20–40 km offshore that had background concentrations based on long-term measurements (1994–2003) at clean coastal locations (i.e., Lompoc, CA). This daily data was averaged into a single variable representing exposure for the previous month.

Based on available residential locations, geo-code quality was sufficient for assignment of air pollution exposures for 99% of reported addresses. Subjects were excluded if they did not have a properly geo-coded address or were missing air pollution or maternal serum cytokine and chemokine data. In this analysis, 379 children with ASD (of which 180 also had ID), 164 with ID but not ASD, and 414 GP controls (total *N* = 960) were included.

### Statistical methods

Statistical analysis was conducted using the R package (www.r-project.org). To characterize the relationship between maternal cytokine and chemokine levels measured during mid-pregnancy and previous month (short term) air pollution exposure, we calculated Spearman correlations between each air pollutant (e.g., PM_10_ and PM_2.5_) and each cytokine and chemokine (e.g., IL-1β and MCP-1). We used analysis of covariance (ANCOVA) to identify any significant mean difference in the covariation of air pollutant exposure and immune marker across the four diagnostic groups.

**Fig. 1:**
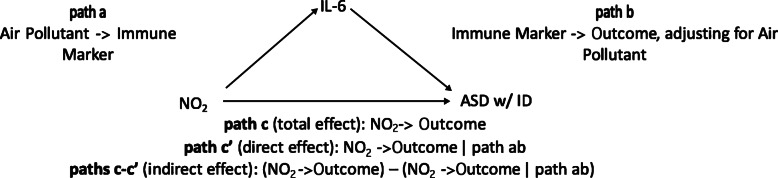
Mediation analysis examined in the Early Markers for Autism study

To assess whether mid-pregnancy immune markers mediate a relationship between mid-pregnancy air pollutant exposure and neurodevelopmental disorders, we tested each possible relationship, as depicted in Fig. 1. We first performed logistic regression models between each air pollution exposure and each neurodevelopmental disorder (relative to GP), adjusting for child sex, year and month of birth, county (Imperial and Orange county were collapsed), and insurance type at the time of birth (Fig. 1, path c). The first three of these variables were included in the EMA design, and the second two (county and insurance type at the time of birth) were included to attempt to account for demographic factors which may influence air pollution-disorder relationships. Immune markers were natural log transformed for all regression analyses.

Next, we used linear regression models to examine the relationship between individual air pollutant exposure and each immune marker in order to estimate the association between the predictor and potential mediator (Fig. 1, path a). Again, regression models were adjusted for child sex, year and month of birth, county (Imperial and Orange county were collapsed), and insurance type. Models in which there was a significant (*p* < 0.001) association between the air pollutant (exposure) and immune marker (outcome) were treated as candidates for the mediation analysis. We then performed linear regression models estimating the association between the candidate immune marker and neurodevelopmental disorder, adjusting for the candidate air pollutant (Fig. 1, path b). This allows us to estimate the relationship between the immune maker and diagnosis, removing any possible confounding by air pollution. Lastly, we used natural effects modeling (R package “medflex”) to estimate the total (Fig. 1, path c), direct (Fig. 1, path c’), and indirect (Fig. 1, paths c and c’) effects of the candidate mediation models (Fig. 1). We implemented each of the models described above (paths a, b, c, and c’) in three separate analytic datasets which each contained GP controls and individuals with one of the neurodevelopmental outcomes of interest (i.e., ASD with ID, ASD without ID, ID only).

## Results

### Sample characteristics

Overall, EMA mothers predominantly resided in Orange and San Diego counties at the time of birth (45% and 53%, respectively), were of Hispanic ethnicity (46%), and were on average 29 years of age at child’s birth (Table 1). Demographic characteristics differed across child neurodevelopmental groups for all factors examined except paternal age. For example, the proportion of subjects reporting the use of public insurance for prenatal care ranged from 19.6% in the ASD without ID group to 32% in the ASD with ID group and nearly 60% in the ID only group. Mothers of children with ID only reported the highest percent of Hispanic ethnicity (68%) and most frequently resided in San Diego County near the time of birth.

**Table 1:** Sample characteristics of the Early Markers for Autism (EMA) study

	Total (*n* = 957)	ASD without ID (*n* = 199)	ASD with ID (*n* = 180)	ID only (*n* = 164)	GP (*n* = 414)	*p* value
	Mean (SD) or %					
Mom age (years)	28.9 (5.7)	30.3 (5.6)	29.6 (5.8)	27.1 (6.1)	28.7 (5.4)	< 0.01
Dad age (years)	35.1 (15.8)	34.9 (12.6)	36.2 (16.5)	36.6 (21)	34.1 (14.3)	0.26
Birthweight (g)	3371.9 (595.5)	3437 (615)	3404.9 (519.7)	3157.8 (705.6)	3411 (551.2)	< 0.01
Child sex						
Female	21.8	17.6	19.4	41.5	17.1	< 0.01
Male	78.2	82.4	80.6	58.5	82.9	
Maternal race						
White	78.9	77.9	71.7	86.6	79.5	0.04
Asian	11.6	13.1	16.7	5.5	11.1	
Others	9.5	9.0	11.7	7.9	9.4	
Hispanic ethnicity	46.4	32.2	42.2	68.3	46.4	< 0.01
Public insurance	35.6	19.6	32.2	59.8	35.3	< 0.01
County						
Orange County	45.4	42.7	38.9	32.3	54.6	<0.01
San Diego	52.6	56.3	59.4	63.4	43.5	
Imperial	2.1	1.0	1.7	4.3	1.9	

Note: *p* value for ANOVA/chi-squared test for difference across diagnostic groups. *SD* standard deviation

### Correlations among air pollution exposures and cytokines/chemokines

Average exposure levels for the entire study population were 22.1 (8.5 standard deviation (SD)) ppb for NO_2_, 18.3 (5.8 SD) μg/m^3^ for PM_2.5_, 36.8 (11.3 SD) μg/m^3^ for PM_10_, and 38.4 (10.6 SD) ppm for ozone. Pairwise correlations between PM_10_, PM_2.5_, and NO_2_ were moderate to high (*r* = 0.49 for PM_10_ and NO_2_, *r* = 0.73 for PM_2.5_ and NO_2_, *r* = 0.67 for PM_2.5_ and PM_10_). Ozone was inversely correlated with PM_10_, PM_2.5_, and NO_2_ (*r* = − 0.22, *r* = − 0.42, *r* = − 0.72, respectively) (Supplemental Table 1). Measured cytokines and chemokines showed moderate to high correlations with each other; most correlations between analytes were positive (Supplemental Table 2). High correlations (ranging from *r* = 0.70 to *r* = 0.85) were found between IL-6 and IL-1β, IL-8 and IL-1β, IL-8 and IL-6, MIP-1α and IL-1β, MIP-1α and IL-6, and MIP-1α and IL-8. More moderate correlations (*r* = 0.36 to *r* = 0.44) were found between all remaining pairs of maternal cytokines and chemokines.

### Cytokine/chemokine levels and previous month air pollution exposure

Correlations between air pollution exposure and immune analytes in the full EMA sample, regardless of child neurodevelopmental outcome, were generally weak in magnitude (rho < 0.2), although some did reach statistical significance (Table 2). For example, increasing level of NO_2_ was correlated (*p* < 0.05) with decreasing levels of the inflammatory cytokines IL-1α, IL-6, and IL-8; the regulatory cytokine IL-10; the inflammatory chemokine MCP-1; the inflammatory IL-1 receptor antagonist IL-1Ra; and the growth factor GM-CSF. In contrast, the increasing level of PM_2.5_ was correlated (*p* < 0.05) with increasing levels of IL-6, IL-8, and MCP-1. Ozone and PM_10_ showed negative correlations with MCP-1 and the T cell growth factor IL-7, respectively (*p* < 0.05). Follow-up ANCOVA demonstrated significant covariance of all air pollutants with IL-1β, IL-6, IL-8, MCP-1, and MIP-1α (*p* < 0.05).

**Table 2:**
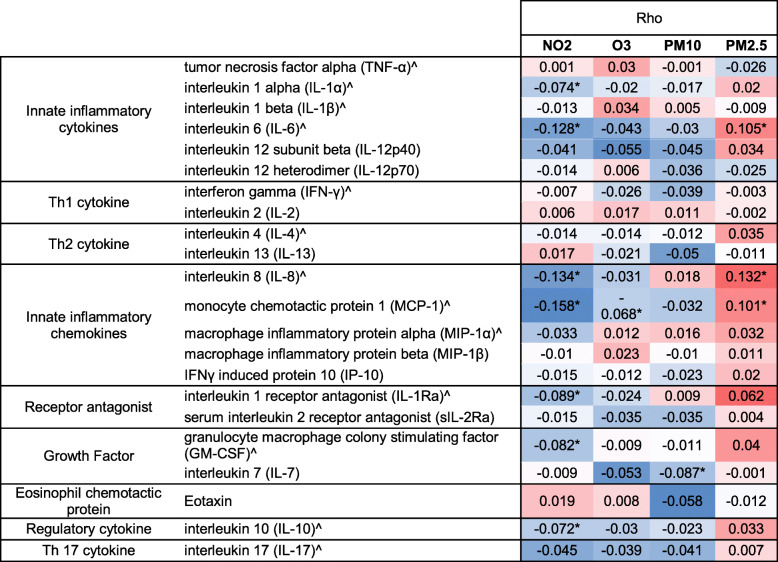
Correlation (Spearman rho) between each measured immune marker and each previous month average air pollution exposure across all diagnostic groups (*n* = 957)

^^^Cytokines/chemokines previously associated with ASD with ID and ID in Jones et al. paper. Shaded cells indicate a positive (red) or negative (blue) correlation. *Significant correlation at *p* = 0.05. Air pollutants measured in parts per billion (NO_2_, ozone) or microgram/meters^3^ (PM_2.5_, PM_10_)

### Mediation analysis

To examine possible mediation of previous month air pollution exposure by immune markers and risk for neurodevelopmental outcomes, we first examined the relationship between each air pollutant and each outcome (ASD without ID, ASD with ID, ID only) as compared to general population controls. We did not find statistically significant relationships between previous month air pollution exposure and any diagnostic group (Table 3).

**Table 3:** Adjusted multivariable logistic regression models estimating odds of diagnosis (ASD with ID, ASD without ID, ID only) given each air pollutant (NO_2_, ozone, PM_10_, PM_2.5_) for a 1 Unit change in exposure

Predictors	Outcome: ASD with ID (vs GP)			Outcome: ASD without ID (vs GP)			Outcome: ID only (vs GP)		
	OR	95% CI	*p*	OR	95% CI	*p*	OR	95% CI	*p*
NO_2_	0.99	(0.97, 1.01)	0.31	1.00	(0.98, 1.02)	0.83	0.99	(0.96, 1.01)	0.34
Child sex (male)	0.88	(0.55, 1.39)	0.58	0.89	(0.56, 1.41)	0.62	0.30	(0.19, 0.45)	< 0.01
Year of birth	0.96	(0.78, 1.18)	0.71	1.04	(0.85, 1.27)	0.69	0.82	(0.65, 1.03)	0.09
Month of birth	1.01	(0.95, 1.08)	0.69	0.98	(0.92, 1.04)	0.56	0.99	(0.92, 1.05)	0.68
SD County (vs Imperial/Orange)	1.82	(1.26, 2.61)	< 0.01	1.63	(1.14, 2.32)	0.01	2.23	(1.49, 3.33)	< 0.01
Public insurance	0.87	(0.60, 1.27)	0.47	0.46	(0.30, 0.69)	< 0.01	2.59	(1.75, 3.83)	< 0.01
	OR	95% CI	*p*	OR	95% CI	*p*	OR	95% CI	*p*
Ozone	1.01	(0.99, 1.02)	0.50	1.00	(0.98, 1.02)	0.95	1.00	(0.98, 1.02)	0.87
Child sex (male)	0.88	(0.55, 1.39)	0.57	0.89	(0.56, 1.41)	0.62	0.29	(0.19, 0.45)	< 0.01
Year of birth	0.97	(0.79, 1.19)	0.76	1.05	(0.86, 1.27)	0.66	0.85	(0.68, 1.06)	0.15
Month of birth	1.02	(0.96, 1.08)	0.52	0.98	(0.93, 1.04)	0.59	1.00	(0.94, 1.07)	0.99
SD County (vs Imperial/Orange)	1.85	(1.29, 2.65)	< 0.01	1.64	(1.16, 2.32)	0.01	2.31	(1.55, 3.44)	< 0.01
Public insurance	0.86	(0.59, 1.26)	0.45	0.46	(0.30, 0.69)	< 0.01	2.57	(1.74, 3.80)	< 0.01
	OR	95% CI	*p*	OR	95% CI	*p*	OR	95% CI	*p*
PM_10_	0.99	(0.97, 1.01)	0.45	0.99	(0.97, 1.01)	0.34	1.02	(1.00, 1.04)	0.07
Child sex (male)	0.86	(0.55, 1.37)	0.53	0.89	(0.56, 1.41)	0.63	0.29	(0.19, 0.45)	< 0.01
Year of birth	0.97	(0.79, 1.19)	0.77	1.03	(0.85, 1.25)	0.77	0.88	(0.70, 1.09)	0.24
Month of birth	1.02	(0.96, 1.08)	0.59	0.98	(0.92, 1.04)	0.42	1.02	(0.95, 1.09)	0.59
SD County (vs Imperial/Orange)	1.72	(1.13, 2.62)	0.01	1.49	(1.00, 2.22)	0.05	2.86	(1.79, 4.55)	< 0.01
Public insurance	0.87	(0.59, 1.26)	0.46	0.46	(0.30, 0.69)	< 0.01	2.54	(1.72, 3.76)	< 0.01
	OR	95% CI	*p*	OR	95% CI	*p*	OR	95% CI	*p*
PM_2.5_	0.97	(0.93, 1.00)	0.06	1.00	(0.96, 1.03)	0.89	0.98	(0.95, 1.02)	0.41
Child sex (male)	0.89	(0.56, 1.41)	0.61	0.89	(0.56, 1.41)	0.62	0.29	(0.19, 0.45)	< 0.01
Year of birth	0.96	(0.78, 1.17)	0.66	1.04	(0.86, 1.27)	0.66	0.83	(0.67, 1.04)	0.10
Month of birth	1.00	(0.94, 1.07)	0.91	0.98	(0.92, 1.05)	0.58	0.99	(0.92, 1.06)	0.73
SD County (vs Imperial/Orange)	1.61	(1.09, 2.38)	0.02	1.62	(1.12, 2.37)	0.01	2.15	(1.41, 3.29)	< 0.01
Public insurance	0.87	(0.6, 1.27)	0.48	0.46	(0.30, 0.69)	< 0.01	2.57	(1.74, 3.80)	< 0.01

Next, as described in Fig. 1, path a, we examined the relationship between air pollution exposure and each immune marker, adjusted for covariates, using separate models for each diagnostic group (ASD with ID, ASD without ID, ID only) compared to general population controls (Supplemental Tables 3-5). A total of ten significant (at *p* < 0.001 threshold) associations were identified and are presented in Table 4. As NO_2_ increased, IL-6 and IL-8 decreased when examining ASD with ID, or ID only, compared to controls (*p* < 0.001). Additional significant relationships were found between increasing NO_2_ and decreasing MCP-1 for ASD without ID and ID only, compared to controls (*p* < 0.001). IL-8 was found to increase as ozone increased for ASD with and ASD without ID. Decreasing IL-10 was associated with increasing NO_2_ (*p* < 0.001) among ID only.

**Table 4:** Adjusted (adjusted for the following covariates: child sex, month and year of birth, county, and insurance type) linear and logistic regression models estimating paths A and B of candidate mediation models

Path A Air pollutant -> immune marker				Path B Immune marker + air pollutant -> outcome				
	Beta	95% CI	*p*			OR	95% CI	*p*
NO_2_ -> IL-6	− 0.05	(− 0.08, − 0.03)	< 0.001	NO_2_ + IL-6 -> ASD w/ ID		0.99	(0.97, 1.02)	0.57
						1.11	(1.03, 1.20)	0.01^a^
NO_2_ -> IL-8	− 0.03	(− 0.05, − 0.02)	< 0.001	NO_2_ + IL-8-> ASD w/ ID		0.99	(0.97, 1.01)	0.44
						1.09	(0.96, 1.24)	0.18
NO_2_ -> MCP-1	− 0.02	(− 0.03, − 0.01)	< 0.001	NO_2_ + MCP-1-> ASD w/ ID		0.99	(0.97, 1.01)	0.40
						1.10	(0.89, 1.36)	0.37
Ozone -> IL-8	0.02	(0.01, 0.03)	< 0.001	Ozone + IL-8-> ASD w/ ID		1.00	(0.99, 1.02)	0.64
						1.09	(0.96, 1.24)	0.16
NO_2_ -> MCP-1	− 0.02	(− 0.02, − 0.01)	< 0.001	NO_2_ + MCP-1-> ASD w/o ID		0.99	(0.97, 1.02)	0.58
						0.77	(0.62, 0.97)	0.03^a^
Ozone -> IL-8	0.02	(0.01, 0.03)	< 0.001	Ozone + IL-8-> ASD w/o ID		1.00	(0.99, 1.02)	0.67
						0.88	(0.78, 0.99)	0.03^a^
NO_2_ -> IL-10	− 0.03	(− 0.05, − 0.02)	< 0.001	NO_2_ + IL-10-> ASD w/o ID		0.99	(0.96, 1.01)	0.32
						0.98	(0.87, 1.10)	0.74
NO_2_ -> IL-6	− 0.05	(− 0.07, − 0.02)	< 0.001	NO_2_ + IL-6-> ID w/o ASD		0.99	(0.96, 1.01)	0.29
						0.97	(0.89, 1.05)	0.45
NO_2_ -> IL-8	− 0.03	(− 0.04, − 0.01)	< 0.001	NO_2_ + IL-8-> ID w/o ASD		0.99	(0.96, 1.01)	0.29
						0.94	(0.82, 1.08)	0.40
NO_2_ -> MCP-1	− 0.02	(− 0.03, − 0.01)	< 0.001	NO_2_ + MCP-1-> ID w/o ASD		0.99	(0.96, 1.01)	0.31
						0.94	(0.74, 1.2)	0.63

Ten candidate mediation models were carried out based on significant associations between air pollutants and immune markers (*p* < 0.001) in supplemental tables 3–5

**Fig. 2:**
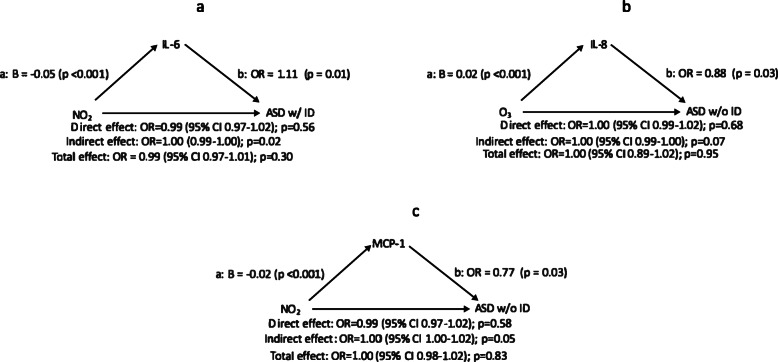
Mediation analysis for three air pollutant and cytokine pairs in the Early Markers for Autism study. **a** Possible mediation by Il-6 for the relationship between NO_2_ and ASD with ID. **b** Possible mediation by Il-8 for the relationship between ozone and ASD without ID. **c** Possible mediation by MCP-1 for the relationship between NO_2_ and ASD without ID

^a^This model is shown in Fig. 2. Air pollutants measured in parts per billion (NO_2_, ozone) or microgram/meters^3^ (PM_2.5_, PM_10_)

For these ten associations, we examined the relationship between the immune marker and the diagnostic outcome, adjusting for the air pollutant exposure, reflecting Fig. 1, path b (Table 4, left column). Here, ASD with ID risk was associated with increasing IL-6, even after adjusting for air pollution exposure, but the other immune marker associations were attenuated (or not significant) with AP adjustment. For ASD without ID, the two immune markers, IL-8 and MCP-1, remained associated after adjusting for ozone and NO_2_, respectively . We did not find that mid-pregnancy air pollution exposure was directly associated with risk for the developmental disorders (Supplemental Tables 6-8). Indirect effects with significant or near significant results were identified in three models (Fig. 2), suggesting that air pollution might act through the immune system. However, the magnitude of this effect was equal to 1.0. Overall, mediation analyses revealed that air pollution exposure was not directly associated with many outcomes, nor did they show that such an effect was mediated by the immune markers. However, immune markers were associated with an increased risk of the outcomes independently of air pollution.

## Discussion

This study found some relationships between maternal serum cytokine and chemokine levels measured mid-gestation and air pollution exposure during the prior month, but no direct effects of AP on ND, or potential mediation of such associations by maternal mid-pregnancy immune response. Thus, we noted that a defined window of exposure to air pollution, reflecting short-term exposure in the early second trimester, was associated with maternal mid-gestational serum levels of several cytokines and chemokines. In particular, greater maternal exposure to air pollutants exhibited varying IL-1β, IL-6, IL-8, MIP-1α, MIP-1β, and MCP-1 levels regardless of neurodevelopmental outcome of the child.

Limited epidemiologic studies have examined the relationship between prenatal air pollution exposure and maternal immune response. Studies of air pollution exposure during pregnancy have predominantly evaluated immune response in the cord blood, or in peripheral samples collected from offspring, but not directly from the mother. Prenatal air pollution exposure to PM_10_ and NO_2_ has been associated with changes in T cell proportion in the cord blood [5]. Other studies showed that exposure to polycyclic aromatic hydrocarbons (PAH) and PM_2.5_ during pregnancy was associated with lower levels of IgE in early pregnancy and higher levels of IgE in later pregnancy [19] and that increasing maternal prenatal NO_2_ exposure was associated with increasing cord blood IL-33 and TSLP [3]. More recent reports showed significantly higher levels of IL-6 in serum of 8-year-old children were associated with NO_2_ exposure during infancy [17]. NO_2_ exposure during infancy was also associated with IL-10 levels among asthmatic children, identifying a potentially susceptible subset of the population [17].

Other studies have capitalized on natural experiments or interventions to investigate the relationship between short-term air pollution exposure and circulating cytokines and chemokines. Comparison of circulating inflammatory markers during and after the Beijing Olympics, when regulations were put in place to reduce vehicular emissions and air quality, showed decreases in several cytokines during the Olympic period including IL-6 and IL-8 [14]. In our study, we found that IL-6 and IL-8 were negatively correlated with NO_2_, but positively correlated with PM_2.5_. While it is not possible to directly compare these findings, the Beijing study, which focused on ultrafine particle assessment, and ours, with measures of PM_2.5_, could be considered roughly consistent.

When exploring the potential role of maternal mid-pregnancy immune markers as mediators in a relationship between air pollution exposure and neurodevelopmental disorders, we first examined immune markers and mid-pregnancy air pollutant levels. We found that several of the immune markers, including IL-6, IL-8, IL-10, and MCP-1, were associated with NO_2_ and/or ozone in subjects with specific diagnoses as compared to general population controls. While no direct effects of mid-pregnancy air pollution exposure on the risk of neurodevelopmental disorders were identified, three statistically significant indirect effects (e.g., mediation by a maternal immune marker) were present. However, these estimates of effect were equal to an OR = 1.0. Possible explanations for this may be antagonistic effects of air pollution on the immune marker, and immune marker on outcome, or type I error. It is also possible that further maternal immune conditions, such as asthma, combine with air pollution to elicit risk.

To our knowledge, this is the first study to examine the association between mid-gestational air pollution exposure and maternal cytokines and chemokines in relation to the risk of having a child with ASD and/or ID. Several epidemiologic studies have suggested that exposure to air pollutants may play a role in the etiology of ASD ([18, 30]. Previous studies in California identified an increased risk of ASD among children whose mothers lived closer to a freeway (a proxy for traffic-related air pollution exposure) around the time of birth [42] as well as higher risk of ASD among children exposed to increasing amounts of traffic-related air pollution, NO_2_, and particulate matter less than 10 and 2.5 microns in diameter (PM_10_ and PM_2.5_) [43]. Other studies from California show increased ASD risk with PM_10_ and hazardous air pollutants, including diesel exhaust particles, solvents, and airborne heavy metals [26, 44]. Notably, these studies examined multiple periods of exposure and most reliably identified associations with late pregnancy or early life, in contrast to the mid-pregnancy assignment here where we found no association between air pollution and any developmental disorder risk. Additionally, these studies examined longer periods of exposure (e.g., trimesters, pregnancy averages) where effect estimates are often standardized to a range (2 standard deviations or inter-quartile range) improving interpretability of effect estimates, and with the benefit of placing those estimates in context with regional or national values. In contrast, our exposure window of 1 month is relatively acute, and as such more difficult to place in the context of regional or national values. Additionally, for a shorter exposure period, we expect a larger range of values to be present. For these reasons, we chose to examine the effect of a per-unit change in exposure, where our estimates of effect might be small, rather than artificially inflated should we employ a scaling metric.

Similarly, an expanding area of research has identified dysregulation of the gestational immune environment as a risk factor for ASD [35]. Elevated levels of circulating innate inflammatory cytokines and chemokines have been observed in individuals with ASD and in their mothers during mid-gestation compared to general population controls (P [15, 24].). In addition, there is an increasing number of studies describing neonatal immune activity as a predictor of ASD [21, 29]. There is evidence of bidirectional circulation of a limited number of inflammatory cytokines between the maternal and fetal compartments [46], suggesting the potential for dysregulated cytokines in the mother during gestation to influence the developing immune system of the newborn [39]. Thus, while not all cytokines/chemokines cross the placenta and directly influence the fetal immune system, there are likely indirect effects of maternal inflammation on the immune system and neurodevelopment of the developing offspring [38]. In support of this concept, animal models of maternal immune activation have demonstrated that perturbations to the maternal immune environment during gestation produce ASD-relevant behaviors in prenatally exposed offspring [12, 35]. The exact mechanism(s) through which circulating maternal cytokines and chemokines affect fetal neurodevelopment remain to be elucidated. However, numerous studies have shown that they play critical roles in the normal development of the central nervous system, including neurogenesis, neuronal and glial cell migration, proliferation, differentiation, and synaptic maturation and pruning [6, 11]. Components of the immune system, including circulating maternal cytokines and chemokines, have additionally been demonstrated to be essential for the establishment of the placenta and maintenance of normal pregnancies [2, 10]. Therefore, alterations to the gestational immune environment may lead to alterations in the neurodevelopmental trajectory of the developing fetus, potentially resulting in an altered behavioral phenotype characteristic of children with ASD.

This study has several limitations. The maternal immune markers evaluated here were only measured at a single time point in pregnancy, limiting our ability to evaluate how the maternal immune response throughout gestation might affect child development. As a result of this single time point collection, our air pollution exposure was tied in time most closely to the date of serum sampling and may not reflect other critical periods of exposure found to be associated with ASD in other studies [25, 34, 43]. While we evaluated associations with multiple outcome groups to examine specificity of associations, this resulted in a large number of statistical tests. Statistical methods to reduce type I error might be needed for future investigations; however, our goal was to evaluate novel relationships of mediation and mid-pregnancy exposure-immune correspondence and we thus chose to employ nominal significance levels. Post doc examination of power for our ANCOVA resulted in over 90% power to detect an overall effect, given the full sample size in Table 1, a 4-group design, and a two-level covariate. Additionally, post doc estimation of single air pollutant to immune marker relationship indicated 80% power to detect an effect of 0.05 or greater at group sample sizes used in our analysis. However, these calculations do not account for a multiple comparison correction, like Bonferroni, for which calculated powers were approximately half. Additionally, EMA was designed as a case-control study though common quantitative measures (here, maternal immune markers) are present across all possible recruitment groups. We caution that initial evaluation of previous month air pollution exposure and maternal immune markers may be enriched by the increased presence of subjects with neurodevelopmental disorders and may not be generalizable to all pregnancy measures in the population. Findings present in the general population controls might be more indicative of broad population effects. Despite these limitations, this study begins to address a critical gap in how maternal immune system activation by environmental exposures during pregnancy might affect offspring neurodevelopment.

## Conclusions

This study suggests that maternal immune activation is associated with risk for neurodevelopemental disorders. Furthermore, that prenatal air pollution exposure is associated with small, but perhaps biologically relevant, effects on maternal immune system function during pregnancy. Further studies are needed to evaluate how prenatal exposure to air pollution affects the trajectory of maternal immune activation during pregnancy, if windows of heightened susceptibility can be identified, and how these factors influence neurodevelopment of the offspring.

## Supplementary Information

**Additional file 1: Supplemental Table 1**. Correlation (Spearman Rho) between each air pollutant. **Supplemental Table 2**. Correlation (Spearman Rho) between each measured immune marker. **Supplemental Table 3**. Linear Regression Models: ln (Immune Marker) ~ Air Pollutant + Covariates, among ASD w/ ID + GP analytic dataset. **Supplemental Table 4**. Linear Regression Models: ln (Immune Marker) ~ Air Pollutant + Covariates, among ASD wo/ ID + GP analytic dataset. **Supplemental Table 5**. Linear Regression Models: ln (Immune Marker) ~ Air Pollutant + Covariates, among ID w/o ASD + GP analytic dataset. **Supplemental Table 6**. Natural Effects Mediation Modeling to assess whether immune marker mediates association between air pollutant and ASD w/ ID (relative to GP). **Supplemental Table 7**. Natural Effects Mediation Modeling to assess whether immune marker mediates association between air pollutant and ASD w/o ID (relative to GP). **Supplemental Table 8**. Natural Effects Mediation Modeling to assess whether immune marker mediates association between air pollutant and ID w/o (relative to GP).

## Data Availability

Not applicable
