# Supplementary material for: Maternal immune response and air pollution exposure during pregnancy: insights from the Early Markers for Autism (EMA) study
Source: J Neurodev Disord. 2020 Dec 16;12:42. doi: 10.1186/s11689-020-09343-0 (PMC7745402; doi:10.1186/s11689-020-09343-0)
Supplement: Supplementary file 1 — Additional file 1: Supplemental Table 1. Correlation (Spearman Rho) between each air pollutant. Supplemental Table 2. Correlation (Spearman Rho) between each measured immune marker. Supplemental Table 3. Linear Regression Models: ln (Immune Marker) ~ Air Pollutant + Covariates, among ASD w/ ID + GP analytic dataset. Supplemental Table 4. Linear Regression Models: ln (Immune Marker) ~ Air Pollutant + Covariates, among ASD wo/ ID + GP analytic dataset. Supplemental Table 5. Linear Regression Models: ln (Immune Marker) ~ Air Pollutant + Covariates, among ID w/o ASD + GP analytic dataset. Supplemental Table 6. Natural Effects Mediation Modeling to assess whether immune marker mediates association between air pollutant and ASD w/ ID (relative to GP). Supplemental Table 7. Natural Effects Mediation Modeling to assess whether immune marker mediates association between air pollutant and ASD w/o ID (relative to GP). Supplemental Table 8. Natural Effects Mediation Modeling to assess whether immune marker mediates association between air pollutant and ID w/o (relative to GP). [file 11689_2020_9343_MOESM1_ESM.docx]

Supplemental Table 1. Correlation (Spearman Rho) between each air pollutant.

| Pollutant | NO_2_ | O_3_ | PM10 | PM25 |
| --- | --- | --- | --- | --- |
| NO_2_ | 1.00 | -0.72 | 0.49 | 0.73 |
| O_3_ |  | 1.00 | -0.22 | -0.48 |
| PM10 |  |  | 1.00 | 0.67 |
| PM25 |  |  |  | 1.00 |

Supplemental Table 2. Correlation (Spearman Rho) between each measured immune marker.

|  | Eotaxin | GM-CSF^ | TNF-α^ | IL-1α^ | IL-1β^ | IL-6^ | IFN-γ^ | IL-4^ | IL-10^ | IL-17^ | IL-1Ra^ | IL-8^ | MCP-1^ | MIP-1α^ | IL-2 | IL-7 | IL-13 | IL-12p40 | IL-12p70 | IP-10 | MIP-1β | sIL-2Rα |
| --- | --- | --- | --- | --- | --- | --- | --- | --- | --- | --- | --- | --- | --- | --- | --- | --- | --- | --- | --- | --- | --- | --- |
| Eotaxin | 1 | 0.26 | 0.26 | 0.22 | 0.13 | 0.12 | 0.24 | 0.01 | 0.21 | 0.26 | 0.28 | 0.08 | 0.24 | 0.15 | 0.27 | 0.15 | 0.21 | 0.18 | 0.23 | 0.17 | 0.22 | 0.08 |
| GM-CSF^ |  | 1 | 0.53 | 0.39 | 0.56 | 0.59 | 0.21 | -0.08 | 0.35 | 0.19 | 0.42 | 0.53 | 0.43 | 0.55 | 0.25 | -0.10 | 0.12 | 0.07 | 0.21 | 0.10 | 0.50 | -0.05 |
| TNF-α^ |  |  | 1 | 0.37 | 0.70 | 0.70 | 0.22 | 0.03 | 0.25 | 0.19 | 0.40 | 0.55 | 0.38 | 0.71 | 0.18 | -0.06 | 0.02 | 0.01 | 0.22 | 0.17 | 0.67 | 0.05 |
| IL-1α^ |  |  |  | 1 | 0.44 | 0.40 | 0.21 | 0.01 | 0.52 | 0.18 | 0.64 | 0.27 | 0.21 | 0.34 | 0.27 | 0.27 | 0.49 | 0.39 | 0.25 | 0.11 | 0.37 | 0.07 |
| IL-1β^ |  |  |  |  | 1 | 0.86 | 0.13 | -0.02 | 0.37 | 0.08 | 0.55 | 0.70 | 0.36 | 0.84 | 0.12 | -0.002 | 0.10 | 0.11 | 0.11 | 0.02 | 0.70 | 0.01 |
| IL-6^ |  |  |  |  |  | 1 | 0.17 | 0.01 | 0.30 | 0.12 | 0.48 | 0.76 | 0.38 | 0.84 | 0.13 | -0.05 | 0.05 | -0.01 | 0.15 | 0.05 | 0.70 | 0.00 |
| IFN-γ^ |  |  |  |  |  |  | 1 | -0.02 | 0.26 | 0.62 | 0.19 | 0.08 | 0.11 | 0.15 | 0.32 | 0.16 | 0.24 | 0.12 | 0.37 | 0.07 | 0.21 | 0.05 |
| IL-4^ |  |  |  |  |  |  |  | 1 | -0.04 | 0.02 | 0.00 | 0.00 | -0.02 | -0.04 | 0.02 | 0.04 | -0.04 | 0.03 | 0.10 | 0.06 | 0.00 | 0.16 |
| IL-10^ |  |  |  |  |  |  |  |  | 1 | 0.18 | 0.68 | 0.19 | 0.27 | 0.26 | 0.20 | 0.40 | 0.49 | 0.48 | 0.18 | 0.06 | 0.26 | 0.08 |
| IL-17^ |  |  |  |  |  |  |  |  |  | 1 | 0.15 | 0.04 | 0.07 | 0.10 | 0.33 | 0.13 | 0.20 | 0.14 | 0.40 | 0.05 | 0.18 | 0.07 |
| IL-1Ra^ |  |  |  |  |  |  |  |  |  |  | 1 | 0.43 | 0.37 | 0.44 | 0.24 | 0.33 | 0.41 | 0.52 | 0.21 | 0.08 | 0.42 | 0.08 |
| IL-8^ |  |  |  |  |  |  |  |  |  |  |  | 1 | 0.44 | 0.74 | 0.07 | -0.20 | -0.04 | -0.06 | 0.08 | 0.01 | 0.55 | -0.05 |
| MCP-1^ |  |  |  |  |  |  |  |  |  |  |  |  | 1 | 0.41 | 0.13 | -0.02 | -0.03 | 0.03 | 0.10 | 0.13 | 0.36 | 0.04 |
| MIP-1α^ |  |  |  |  |  |  |  |  |  |  |  |  |  | 1 | 0.10 | -0.11 | 0.00 | -0.03 | 0.13 | 0.05 | 0.80 | -0.02 |
| IL-2 |  |  |  |  |  |  |  |  |  |  |  |  |  |  | 1 | 0.12 | 0.20 | 0.21 | 0.32 | 0.07 | 0.16 | 0.10 |
| IL-7 |  |  |  |  |  |  |  |  |  |  |  |  |  |  |  | 1 | 0.47 | 0.55 | 0.22 | 0.08 | -0.04 | 0.19 |
| IL-13 |  |  |  |  |  |  |  |  |  |  |  |  |  |  |  |  | 1 | 0.50 | 0.25 | 0.01 | 0.07 | 0.08 |
| IL-12p40 |  |  |  |  |  |  |  |  |  |  |  |  |  |  |  |  |  | 1 | 0.21 | 0.01 | 0.03 | 0.16 |
| IL-12p70 |  |  |  |  |  |  |  |  |  |  |  |  |  |  |  |  |  |  | 1 | 0.14 | 0.22 | 0.16 |
| IP-10 |  |  |  |  |  |  |  |  |  |  |  |  |  |  |  |  |  |  |  | 1 | 0.13 | 0.13 |
| MIP-1β |  |  |  |  |  |  |  |  |  |  |  |  |  |  |  |  |  |  |  |  | 1 | -0.01 |
| sIL-2Rα |  |  |  |  |  |  |  |  |  |  |  |  |  |  |  |  |  |  |  |  |  | 1 |

Supplemental Table 3. Linear Regression Models: ln (Immune Marker) ~ Air Pollutant + Covariates, among ASD w/ ID + GP analytic dataset.

| **Immune Marker** | **Air Pollutant** | **Beta** | **95% CI** | **P** |
| --- | --- | --- | --- | --- |
| Eotaxin | NO2 | 0.00 | (-0.02 , 0.01) | 0.65 |
| Eotaxin | O3 | 0.00 | (-0.01 , 0.01) | 0.64 |
| Eotaxin | PM10 | -0.02 | (-0.03 , -0.01) | 0.00 |
| Eotaxin | PM2.5 | 0.00 | (-0.03 , 0.02) | 0.72 |
| GM-CSF | NO2 | -0.03 | (-0.06 , -0.01) | 0.01 |
| GM-CSF | O3 | 0.02 | (0.00 , 0.04) | 0.09 |
| GM-CSF | PM10 | -0.02 | (-0.05 , 0.00) | 0.04 |
| GM-CSF | PM2.5 | -0.01 | (-0.05 , 0.03) | 0.56 |
| IFN-γ | NO2 | -0.01 | (-0.03 , 0.01) | 0.24 |
| IFN-γ | O3 | 0.00 | (-0.01 , 0.02) | 0.51 |
| IFN-γ | PM10 | -0.02 | (-0.03 , 0.00) | 0.03 |
| IFN-γ | PM2.5 | -0.02 | (-0.04 , 0.01) | 0.20 |
| IL-10 | NO2 | -0.03 | (-0.05 , -0.01) | <0.01 |
| IL-10 | O3 | 0.01 | (0.00 , 0.03) | 0.07 |
| IL-10 | PM10 | -0.02 | (-0.04 , -0.01) | 0.00 |
| IL-10 | PM2.5 | -0.03 | (-0.06 , 0.00) | 0.04 |
| IL-12p40 | NO2 | 0.00 | (-0.02 , 0.02) | 0.99 |
| IL-12p40 | O3 | 0.00 | (-0.01 , 0.02) | 0.70 |
| IL-12p40 | PM10 | 0.00 | (-0.02 , 0.02) | 0.85 |
| IL-12p40 | PM2.5 | -0.01 | (-0.05 , 0.02) | 0.40 |
| IL-12p70 | NO2 | -0.01 | (-0.03 , 0.01) | 0.17 |
| IL-12p70 | O3 | 0.00 | (-0.01 , 0.02) | 0.72 |
| IL-12p70 | PM10 | -0.02 | (-0.03 , 0.00) | 0.06 |
| IL-12p70 | PM2.5 | -0.01 | (-0.03 , 0.02) | 0.64 |
| IL-13 | NO2 | 0.01 | (-0.02 , 0.03) | 0.51 |
| IL-13 | O3 | 0.00 | (-0.02 , 0.01) | 0.73 |
| IL-13 | PM10 | -0.01 | (-0.03 , 0.01) | 0.20 |
| IL-13 | PM2.5 | -0.01 | (-0.05 , 0.03) | 0.58 |
| IL-17 | NO2 | -0.02 | (-0.04 , 0.01) | 0.18 |
| IL-17 | O3 | 0.01 | (-0.01 , 0.03) | 0.30 |
| IL-17 | PM | -0.01 | (-0.04 , 0.01) | 0.22 |
| IL-17 | PM2.5 | -0.02 | (-0.05 , 0.02) | 0.38 |
| IL-1b | NO2 | -0.02 | (-0.04 , 0.01) | 0.13 |
| IL-1b | O3 | 0.00 | (-0.01 , 0.02) | 0.79 |
| IL-1b | PM10 | -0.02 | (-0.04 , 0.00) | 0.07 |
| IL-1b | PM2.5 | 0.00 | (-0.04 , 0.03) | 0.88 |
| IL-1ra | NO2 | -0.02 | (-0.03 , 0.00) | 0.01 |
| IL-1ra | O3 | 0.01 | (0.00 , 0.02) | 0.08 |
| IL-1ra | PM10 | -0.01 | (-0.02 , 0.00) | 0.23 |
| IL-1ra | PM2.5 | -0.01 | (-0.03 , 0.01) | 0.35 |
| IL-2 | NO2 | 0.00 | (-0.01 , 0.02) | 0.82 |
| IL-2 | O3 | 0.00 | (-0.01 , 0.01) | 1.00 |
| IL-2 | PM10 | 0.00 | (-0.01 , 0.02) | 0.87 |
| IL-2 | PM2.5 | 0.01 | (-0.01 , 0.04) | 0.26 |
| IL-4 | NO2 | 0.00 | (-0.01 , 0.01) | 0.43 |
| IL-4 | O3 | 0.00 | (0.00 , 0.01) | 0.40 |
| IL-4 | PM10 | 0.00 | (-0.01 , 0.01) | 0.77 |
| IL-4 | PM2.5 | -0.01 | (-0.02 , 0.01) | 0.49 |
| IL-6 | NO2 | -0.05 | (-0.08 , -0.03) | <0.001 |
| IL-6 | O3 | 0.03 | (0.01 , 0.04) | <0.01 |
| IL-6 | PM10 | -0.03 | (-0.05 , -0.01) | 0.01 |
| IL-6 | PM2.5 | -0.03 | (-0.07 , 0.01) | 0.10 |
| IL-7 | NO2 | 0.00 | (-0.01 , 0.01) | 0.93 |
| IL-7 | O3 | 0.00 | (-0.01 , 0.01) | 0.93 |
| IL-7 | PM10 | -0.01 | (-0.02 , 0.00) | 0.20 |
| IL-7 | PM2.5 | 0.00 | (-0.02 , 0.01) | 0.75 |
| IL-8 | NO2 | -0.03 | (-0.05 , -0.02) | <0.001 |
| IL-8 | O3 | 0.02 | (0.01 , 0.03) | <0.001 |
| IL-8 | PM10 | -0.02 | (-0.03 , 0.00) | 0.01 |
| IL-8 | PM2.5 | -0.02 | (-0.05 , 0.00) | 0.05 |
| IL-1a | NO2 | -0.03 | (-0.05 , 0.00) | 0.02 |
| IL-1a | O3 | 0.01 | (-0.01 , 0.03) | 0.30 |
| IL-1a | PM10 | -0.02 | (-0.04 , 0.00) | 0.06 |
| IL-1a | PM2.5 | -0.01 | (-0.05 , 0.02) | 0.42 |
| IP-10 | NO2 | 0.00 | (-0.01 , 0.01) | 0.95 |
| IP-10 | O3 | 0.00 | (-0.01 , 0.01) | 0.84 |
| IP-10 | PM10 | 0.00 | (-0.01 , 0.00) | 0.23 |
| IP-10 | PM2.5 | 0.00 | (-0.01 , 0.01) | 0.87 |
| lnmcp_1 | NO2 | -0.02 | (-0.03 , -0.01) | <0.001 |
| lnmcp_1 | O3 | 0.01 | (0.00 , 0.02) | 0.01 |
| lnmcp_1 | PM10 | -0.01 | (-0.02 , 0.00) | 0.07 |
| lnmcp_1 | PM2.5 | -0.01 | (-0.03 , 0.00) | 0.06 |
| MIP-1α | NO2 | -0.02 | (-0.05 , 0.00) | 0.10 |
| MIP-1α | O3 | 0.01 | (-0.01 , 0.03) | 0.44 |
| MIP-1α | PM10 | -0.02 | (-0.04 , 0.00) | 0.06 |
| MIP-1α | PM2.5 | -0.01 | (-0.05 , 0.03) | 0.63 |
| MIP-1β | NO2 | -0.01 | (-0.02 , 0.01) | 0.31 |
| MIP-1β | O3 | 0.00 | (-0.01 , 0.02) | 0.41 |
| MIP-1β | PM10 | -0.01 | (-0.03 , 0.00) | 0.04 |
| MIP-1β | PM2.5 | 0.00 | (-0.02 , 0.02) | 0.95 |
| sIL-2Ra | NO2 | -0.01 | (-0.02 , 0.01) | 0.25 |
| sIL-2Ra | O3 | 0.00 | (-0.01 , 0.01) | 0.67 |
| sIL-2Ra | PM10 | 0.00 | (-0.01 , 0.01) | 0.61 |
| sIL-2Ra | PM2.5 | -0.01 | (-0.03 , 0.00) | 0.14 |
| TNF-α | NO2 | -0.01 | (-0.02 , 0.01) | 0.30 |
| TNF-α | O3 | 0.00 | (-0.01 , 0.01) | 0.89 |
| TNF-α | PM10 | -0.01 | (-0.02 , 0.00) | 0.19 |
| TNF-α | PM2.5 | 0.00 | (-0.02 , 0.02) | 0.97 |

Supplemental Table 4. Linear Regression Models: ln (Immune Marker) ~ Air Pollutant + Covariates, among ASD wo/ ID + GP analytic dataset.

| **Immune Marker** | **Air Pollutant** | **Beta** | **95% CI** | **P** |
| --- | --- | --- | --- | --- |
| Eotaxin | NO2 | 0.00 | (-0.02 , 0.01) | 0.80 |
| Eotaxin | O3 | 0.01 | (0.00 , 0.02) | 0.26 |
| Eotaxin | PM10 | -0.01 | (-0.02 , 0.00) | 0.12 |
| Eotaxin | PM2.5 | 0.00 | (-0.02 , 0.02) | 0.91 |
| GM-CSF | NO2 | -0.02 | (-0.05 , 0.00) | 0.07 |
| GM-CSF | O3 | 0.01 | (0.00 , 0.03) | 0.15 |
| GM-CSF | PM10 | -0.01 | (-0.04 , 0.01) | 0.20 |
| GM-CSF | PM2.5 | -0.01 | (-0.05 , 0.03) | 0.59 |
| IFN-γ | NO2 | -0.01 | (-0.02 , 0.01) | 0.47 |
| IFN-γ | O3 | 0.00 | (-0.01 , 0.02) | 0.49 |
| IFN-γ | PM10 | -0.01 | (-0.03 , 0.00) | 0.08 |
| IFN-γ | PM2.5 | -0.02 | (-0.04 , 0.01) | 0.18 |
| IL-10 | NO2 | -0.03 | (-0.04 , -0.01) | <0.001 |
| IL-10 | O3 | 0.01 | (0.00 , 0.02) | 0.19 |
| IL-10 | PM10 | -0.02 | (-0.04 , 0.00) | 0.01 |
| IL-10 | PM2.5 | -0.02 | (-0.05 , 0.00) | 0.11 |
| IL-12p40 | NO2 | -0.01 | (-0.03 , 0.01) | 0.28 |
| IL-12p40 | O3 | 0.01 | (-0.01 , 0.03) | 0.19 |
| IL-12p40 | PM10 | 0.00 | (-0.02 , 0.02) | 0.94 |
| IL-12p40 | PM2.5 | -0.02 | (-0.05 , 0.01) | 0.18 |
| IL-12p70 | NO2 | -0.01 | (-0.03 , 0.01) | 0.24 |
| IL-12p70 | O3 | 0.00 | (-0.01 , 0.02) | 0.54 |
| IL-12p70 | PM10 | -0.01 | (-0.03 , 0.00) | 0.07 |
| IL-12p70 | PM2.5 | -0.01 | (-0.04 , 0.01) | 0.28 |
| IL-13 | NO2 | -0.01 | (-0.03 , 0.02) | 0.55 |
| IL-13 | O3 | 0.01 | (-0.01 , 0.02) | 0.53 |
| IL-13 | PM10 | -0.02 | (-0.04 , 0.00) | 0.08 |
| IL-13 | PM2.5 | -0.03 | (-0.06 , 0.01) | 0.13 |
| IL-17 | NO2 | -0.02 | (-0.04 , 0.01) | 0.15 |
| IL-17 | O3 | 0.01 | (0.00 , 0.03) | 0.15 |
| IL-17 | PM10 | -0.01 | (-0.03 , 0.01) | 0.29 |
| IL-17 | PM2.5 | -0.04 | (-0.08 , -0.01) | 0.02 |
| IL-1b | NO2 | -0.01 | (-0.03 , 0.02) | 0.59 |
| IL-1b | O3 | 0.00 | (-0.02 , 0.02) | 0.96 |
| IL-1b | PM10 | -0.01 | (-0.03 , 0.01) | 0.50 |
| IL-1b | PM2.5 | 0.03 | (-0.01 , 0.06) | 0.13 |
| IL-1ra | NO2 | -0.01 | (-0.03 , 0.00) | 0.02 |
| IL-1ra | O3 | 0.01 | (0.00 , 0.02) | 0.09 |
| IL-1ra | PM10 | -0.01 | (-0.02 , 0.00) | 0.25 |
| IL-1ra | PM2.5 | -0.01 | (-0.03 , 0.01) | 0.40 |
| IL-2 | NO2 | 0.01 | (-0.01 , 0.02) | 0.52 |
| IL-2 | O3 | 0.00 | (-0.01 , 0.01) | 0.98 |
| IL-2 | PM10 | 0.01 | (0.00 , 0.02) | 0.13 |
| IL-2 | PM2.5 | 0.01 | (-0.02 , 0.03) | 0.52 |
| IL-4 | NO2 | -0.01 | (-0.02 , 0.00) | 0.24 |
| IL-4 | O3 | 0.01 | (0.00 , 0.01) | 0.12 |
| IL-4 | PM10 | 0.00 | (-0.01 , 0.01) | 0.81 |
| IL-4 | PM2.5 | -0.01 | (-0.02 , 0.01) | 0.24 |
| IL-6 | NO2 | -0.04 | (-0.06 , -0.02) | <0.01 |
| IL-6 | O3 | 0.03 | (0.01 , 0.04) | 0.01 |
| IL-6 | PM10 | -0.02 | (-0.04 , 0.00) | 0.08 |
| IL-6 | PM2.5 | -0.01 | (-0.04 , 0.03) | 0.71 |
| IL-7 | NO2 | 0.00 | (-0.01 , 0.01) | 0.68 |
| IL-7 | O3 | 0.00 | (-0.01 , 0.01) | 0.74 |
| IL-7 | PM10 | -0.01 | (-0.02 , 0.00) | 0.24 |
| IL-7 | PM2.5 | 0.00 | (-0.02 , 0.02) | 0.97 |
| IL-8 | NO2 | -0.03 | (-0.04 , -0.01) | <0.01 |
| IL-8 | O3 | 0.02 | (0.01 , 0.03) | <0.001 |
| IL-8 | PM10 | -0.01 | (-0.02 , 0.01) | 0.22 |
| IL-8 | PM2.5 | -0.02 | (-0.04 , 0.01) | 0.19 |
| IL-1a | NO2 | -0.02 | (-0.05 , 0.00) | 0.03 |
| IL-1a | O3 | 0.01 | (-0.01 , 0.03) | 0.19 |
| IL-1a | PM10 | -0.02 | (-0.04 , 0.00) | 0.05 |
| IL-1a | PM2.5 | -0.02 | (-0.06 , 0.01) | 0.17 |
| IP-10 | NO2 | 0.00 | (-0.01 , 0.00) | 0.41 |
| IP-10 | O3 | 0.00 | (0.00 , 0.01) | 0.26 |
| IP-10 | PM10 | 0.00 | (-0.01 , 0.01) | 0.60 |
| IP-10 | PM2.5 | 0.00 | (-0.01 , 0.01) | 0.99 |
| lnmcp_1 | NO2 | -0.02 | (-0.02 , -0.01) | <0.01 |
| lnmcp_1 | O3 | 0.01 | (0.00 , 0.01) | 0.01 |
| lnmcp_1 | PM10 | -0.01 | (-0.01 , 0.00) | 0.06 |
| lnmcp_1 | PM2.5 | -0.01 | (-0.02 , 0.00) | 0.15 |
| MIP-1α | NO2 | -0.01 | (-0.04 , 0.01) | 0.37 |
| MIP-1α | O3 | 0.01 | (-0.01 , 0.03) | 0.38 |
| MIP-1α | PM10 | 0.00 | (-0.03 , 0.02) | 0.74 |
| MIP-1α | PM2.5 | 0.01 | (-0.02 , 0.05) | 0.43 |
| MIP-1β | NO2 | 0.00 | (-0.02 , 0.01) | 0.61 |
| MIP-1β | O3 | 0.00 | (-0.01 , 0.01) | 0.47 |
| MIP-1β | PM10 | -0.01 | (-0.02 , 0.01) | 0.30 |
| MIP-1β | PM2.5 | 0.01 | (-0.01 , 0.03) | 0.51 |
| sIL-2Ra | NO2 | 0.00 | (-0.02 , 0.01) | 0.63 |
| sIL-2Ra | O3 | 0.00 | (-0.01 , 0.01) | 0.88 |
| sIL-2Ra | PM10 | 0.00 | (-0.01 , 0.01) | 0.90 |
| sIL-2Ra | PM2.5 | 0.00 | (-0.02 , 0.01) | 0.64 |
| TNF-α | NO2 | 0.00 | (-0.01 , 0.01) | 0.86 |
| TNF-α | O3 | 0.00 | (-0.01 , 0.01) | 0.86 |
| TNF-α | PM10 | 0.00 | (-0.01 , 0.01) | 0.45 |
| TNF-α | PM2.5 | 0.01 | (0.00 , 0.03) | 0.11 |

Supplemental Table 5. Linear Regression Models: ln (Immune Marker) ~ Air Pollutant + Covariates, among ID w/o ASD + GP analytic dataset.

| **Immune Marker** | **Air Pollutant** | **Beta** | **95% CI** | **P** |
| --- | --- | --- | --- | --- |
| Eotaxin | NO2 | 0.01 | (0.00 , 0.02) | 0.18 |
| Eotaxin | O3 | 0.00 | (-0.01 , 0.01) | 0.73 |
| Eotaxin | PM10 | -0.01 | (-0.02 , 0.00) | 0.06 |
| Eotaxin | PM2.5 | 0.02 | (0.00 , 0.04) | 0.11 |
| GM-CSF | NO2 | -0.04 | (-0.06 , -0.01) | 0.00 |
| GM-CSF | O3 | 0.02 | (0.00 , 0.04) | 0.02 |
| GM-CSF | PM10 | -0.02 | (-0.04 , 0.00) | 0.05 |
| GM-CSF | PM2.5 | -0.02 | (-0.06 , 0.01) | 0.24 |
| IFN-γ | NO2 | 0.00 | (-0.02 , 0.01) | 0.58 |
| IFN-γ | O3 | 0.01 | (-0.01 , 0.02) | 0.40 |
| IFN-γ | PM10 | -0.01 | (-0.03 , 0.00) | 0.06 |
| IFN-γ | PM2.5 | -0.01 | (-0.04 , 0.01) | 0.39 |
| IL-10 | NO2 | -0.03 | (-0.05 , -0.02) | <0.001 |
| IL-10 | O3 | 0.02 | (0.00 , 0.03) | 0.01 |
| IL-10 | PM10 | -0.02 | (-0.03 , -0.01) | 0.01 |
| IL-10 | PM2.5 | -0.04 | (-0.06 , -0.01) | 0.01 |
| IL-12p40 | NO2 | -0.01 | (-0.03 , 0.01) | 0.28 |
| IL-12p40 | O3 | 0.01 | (0.00 , 0.03) | 0.16 |
| IL-12p40 | PM10 | 0.00 | (-0.02 , 0.02) | 0.92 |
| IL-12p40 | PM2.5 | -0.02 | (-0.05 , 0.01) | 0.14 |
| IL-12p70 | NO2 | -0.01 | (-0.02 , 0.01) | 0.49 |
| IL-12p70 | O3 | 0.00 | (-0.01 , 0.01) | 0.98 |
| IL-12p70 | PM10 | -0.02 | (-0.03 , -0.01) | 0.01 |
| IL-12p70 | PM2.5 | 0.00 | (-0.03 , 0.02) | 0.87 |
| IL-13 | NO2 | 0.00 | (-0.03 , 0.02) | 0.67 |
| IL-13 | O3 | 0.01 | (-0.01 , 0.02) | 0.45 |
| IL-13 | PM10 | -0.02 | (-0.04 , 0.00) | 0.03 |
| IL-13 | PM2.5 | -0.03 | (-0.06 , 0.00) | 0.08 |
| IL-17 | NO2 | 0.00 | (-0.03 , 0.02) | 0.73 |
| IL-17 | O3 | 0.01 | (-0.01 , 0.03) | 0.34 |
| IL-17 | PM10 | -0.01 | (-0.03 , 0.01) | 0.46 |
| IL-17 | PM2.5 | -0.02 | (-0.05 , 0.02) | 0.40 |
| IL-1b | NO2 | -0.01 | (-0.03 , 0.01) | 0.30 |
| IL-1b | O3 | 0.00 | (-0.01 , 0.02) | 0.77 |
| IL-1b | PM10 | -0.01 | (-0.02 , 0.01) | 0.46 |
| IL-1b | PM2.5 | 0.01 | (-0.02 , 0.04) | 0.56 |
| IL-1ra | NO2 | -0.02 | (-0.03 , 0.00) | 0.01 |
| IL-1ra | O3 | 0.01 | (0.00 , 0.02) | 0.01 |
| IL-1ra | PM10 | 0.00 | (-0.01 , 0.01) | 0.59 |
| IL-1ra | PM2.5 | -0.01 | (-0.03 , 0.01) | 0.34 |
| IL-2 | NO2 | 0.01 | (-0.01 , 0.02) | 0.38 |
| IL-2 | O3 | 0.00 | (-0.01 , 0.02) | 0.53 |
| IL-2 | PM10 | 0.00 | (-0.01 , 0.02) | 0.56 |
| IL-2 | PM2.5 | 0.01 | (-0.01 , 0.03) | 0.39 |
| IL-4 | NO2 | -0.01 | (-0.02 , 0.00) | 0.21 |
| IL-4 | O3 | 0.00 | (0.00 , 0.01) | 0.28 |
| IL-4 | PM10 | 0.00 | (-0.01 , 0.01) | 0.93 |
| IL-4 | PM2.5 | -0.01 | (-0.02 , 0.01) | 0.40 |
| IL-6 | NO2 | -0.05 | (-0.07 , -0.02) | <0.001 |
| IL-6 | O3 | 0.03 | (0.01 , 0.05) | <0.01 |
| IL-6 | PM10 | -0.02 | (-0.04 , 0.00) | 0.08 |
| IL-6 | PM2.5 | -0.03 | (-0.06 , 0.01) | 0.16 |
| IL-7 | NO2 | 0.00 | (-0.01 , 0.01) | 0.94 |
| IL-7 | O3 | 0.00 | (-0.01 , 0.01) | 0.88 |
| IL-7 | PM10 | 0.00 | (-0.01 , 0.00) | 0.37 |
| IL-7 | PM2.5 | -0.01 | (-0.02 , 0.01) | 0.43 |
| IL-8 | NO2 | -0.03 | (-0.04 , -0.01) | <0.001 |
| IL-8 | O3 | 0.02 | (0.01 , 0.03) | <0.01 |
| IL-8 | PM10 | -0.01 | (-0.02 , 0.00) | 0.24 |
| IL-8 | PM2.5 | -0.02 | (-0.04 , 0.00) | 0.10 |
| IL-1a | NO2 | -0.02 | (-0.05 , 0.00) | 0.05 |
| IL-1a | O3 | 0.01 | (-0.01 , 0.03) | 0.22 |
| IL-1a | PM10 | -0.02 | (-0.04 , 0.00) | 0.07 |
| IL-1a | PM2.5 | -0.02 | (-0.05 , 0.02) | 0.37 |
| IP-10 | NO2 | 0.00 | (-0.01 , 0.01) | 0.60 |
| IP-10 | O3 | 0.00 | (-0.01 , 0.00) | 0.43 |
| IP-10 | PM10 | 0.00 | (-0.01 , 0.00) | 0.26 |
| IP-10 | PM2.5 | 0.00 | (-0.01 , 0.01) | 0.50 |
| lnmcp_1 | NO2 | -0.02 | (-0.03 , -0.01) | <0.001 |
| lnmcp_1 | O3 | 0.01 | (0.01 , 0.02) | <0.01 |
| lnmcp_1 | PM10 | 0.00 | (-0.01 , 0.00) | 0.33 |
| lnmcp_1 | PM2.5 | -0.01 | (-0.02 , 0.01) | 0.25 |
| MIP-1α | NO2 | -0.02 | (-0.04 , 0.01) | 0.19 |
| MIP-1α | O3 | 0.01 | (-0.01 , 0.03) | 0.36 |
| MIP-1α | PM10 | -0.01 | (-0.03 , 0.01) | 0.50 |
| MIP-1α | PM2.5 | 0.00 | (-0.03 , 0.04) | 0.88 |
| MIP-1β | NO2 | -0.01 | (-0.02 , 0.01) | 0.25 |
| MIP-1β | O3 | 0.01 | (-0.01 , 0.02) | 0.33 |
| MIP-1β | PM10 | -0.01 | (-0.02 , 0.00) | 0.24 |
| MIP-1β | PM2.5 | 0.01 | (-0.01 , 0.03) | 0.56 |
| sIL-2Ra | NO2 | -0.01 | (-0.02 , 0.01) | 0.33 |
| sIL-2Ra | O3 | 0.00 | (-0.01 , 0.01) | 0.87 |
| sIL-2Ra | PM10 | 0.00 | (-0.01 , 0.01) | 0.57 |
| sIL-2Ra | PM2.5 | -0.01 | (-0.02 , 0.01) | 0.58 |
| TNF-α | NO2 | 0.00 | (-0.01 , 0.01) | 0.62 |
| TNF-α | O3 | 0.00 | (-0.01 , 0.01) | 0.79 |
| TNF-α | PM10 | 0.00 | (-0.01 , 0.01) | 0.61 |
| TNF-α | PM2.5 | 0.01 | (-0.01 , 0.03) | 0.27 |

Supplemental Table 6. Natural Effects Mediation Modeling to assess whether immune marker mediates association between air pollutant and ASD w/ ID (relative to GP).

| Outcome | Air Pollutant (predictor) | Immune Marker (mediator) | Effect Estimated | OR | 95% CI | p-value |
| --- | --- | --- | --- | --- | --- | --- |
| ASD w/ ID | NO2 | ln IL-6 | natural direct effect | 0.99 | (0.97 , 1.02) | 0.56 |
| ASD w/ ID | NO2 | ln IL-6 | natural indirect effect | 1.00 | (0.99 , 1.00) | 0.02^c^ |
| ASD w/ ID | NO2 | ln IL-6 | total effect | 0.99 | (0.97 , 1.01) | 0.30 |
| ASD w/ ID | NO2 | ln IL-8 | natural direct effect | 0.99 | (0.97 , 1.01) | 0.43 |
| ASD w/ ID | NO2 | ln IL-8 | natural indirect effect | 1.00 | (0.99 , 1.00) | 0.20 |
| ASD w/ ID | NO2 | ln IL-8 | total effect | 0.99 | (0.97 , 1.01) | 0.30 |
| ASD w/ ID | NO2 | ln MCP-1 | natural direct effect | 0.99 | (0.97 , 1.01) | 0.39 |
| ASD w/ ID | NO2 | ln MCP-1 | natural indirect effect | 1.00 | (0.99 , 1.00) | 0.40 |
| ASD w/ ID | NO2 | ln MCP-1 | total effect | 0.99 | (0.97 , 1.01) | 0.30 |
| ASD w/ ID | Ozone | ln IL-8 | natural direct effect | 1.00 | (0.99 , 1.02) | 0.63 |
| ASD w/ ID | Ozone | ln IL-8 | natural indirect effect | 1.00 | (1.00 , 1.00) | 0.19 |
| ASD w/ ID | Ozone | ln IL-8 | total effect | 1.01 | (0.99 , 1.02) | 0.50 |

^c^ This model is shown in Figure 2.

Supplemental Table 7. Natural Effects Mediation Modeling to assess whether immune marker mediates association between air pollutant and ASD w/o ID (relative to GP).

| Outcome | Air Pollutant (predictor) | Immune Marker (mediator) | Effect Estimated | OR | 95% CI | p-value |
| --- | --- | --- | --- | --- | --- | --- |
| ASD w/o ID | NO2 | ln MCP-1 | natural direct effect | 0.99 | (0.97 , 1.02) | 0.58 |
| ASD w/o ID | NO2 | ln MCP-1 | natural indirect effect | 1.00 | (1.00 , 1.01) | 0.05^c^ |
| ASD w/o ID | NO2 | ln MCP-1 | total effect | 1.00 | (0.98 , 1.02) | 0.83 |
| ASD w/o ID | Ozone | ln IL-8 | natural direct effect | 1.00 | (0.99 , 1.02) | 0.68 |
| ASD w/o ID | Ozone | ln IL-8 | natural indirect effect | 1.00 | (0.99 , 1.00) | 0.07^c^ |
| ASD w/o ID | Ozone | ln IL-8 | total effect | 1.00 | (0.98 , 1.02) | 0.95 |

^c^ This model is shown in Figure 2.

Supplemental Table 8. Natural Effects Mediation Modeling to assess whether immune marker mediates association between air pollutant and ID w/o (relative to GP).

| Outcome | Air Pollutant (predictor) | Immune Marker (mediator) | Effect Estimated | OR | 95% CI | p-value |
| --- | --- | --- | --- | --- | --- | --- |
| ID w/o ASD | NO2 | ln IL-6 | natural direct effect | 0.99 | (0.96 , 1.01) | 0.31 |
| ID w/o ASD | NO2 | ln IL-6 | natural indirect effect | 1.00 | (1.00 , 1.01) | 0.46 |
| ID w/o ASD | NO2 | ln IL-6 | total effect | 0.99 | (0.96 , 1.01) | 0.36 |
| ID w/o ASD | NO2 | ln IL-8 | natural direct effect | 0.99 | (0.96 , 1.01) | 0.31 |
| ID w/o ASD | NO2 | ln IL-8 | natural indirect effect | 1.00 | (1.00 , 1.01) | 0.44 |
| ID w/o ASD | NO2 | ln IL-8 | total effect | 0.99 | (0.96 , 1.01) | 0.36 |
| ID w/o ASD | NO2 | ln IL-10 | natural direct effect | 0.99 | (0.96 , 1.01) | 0.35 |
| ID w/o ASD | NO2 | ln IL-10 | natural indirect effect | 1.00 | (1.00 , 1.00) | 0.73 |
| ID w/o ASD | NO2 | ln IL-10 | total effect | 0.99 | (0.96 , 1.01) | 0.36 |
| ID w/o ASD | NO2 | ln MCP-1 | natural direct effect | 0.99 | (0.96 , 1.01) | 0.33 |
| ID w/o ASD | NO2 | ln MCP-1 | natural indirect effect | 1.00 | (1.00 , 1.01) | 0.64 |
| ID w/o ASD | NO2 | ln MCP-1 | total effect | 0.99 | (0.96 , 1.01) | 0.36 |
